# Genetic variants in a sodium-dependent vitamin C transporter gene and age-related cataract

**DOI:** 10.1136/bjophthalmol-2018-312257

**Published:** 2018-11-15

**Authors:** Ravilla D Ravindran, Periasamy Sundaresan, Tiruvengada Krishnan, Praveen Vashist, Giovanni Maraini, Vijayan Saravanan, Usha Chakravarthy, Liam Smeeth, Dorothea Nitsch, Ian S Young, Astrid E Fletcher

**Affiliations:** 1 Cataract Services, Aravind Eye Hospital, Madurai, India; 2 Department of Genetics, Dr. G. Venkataswamy Eye Research Institute, Aravind Medical Research Foundation, Madurai, India; 3 Cataract Services, Aravind Eye Hospital, Pondicherry, India; 4 Community Ophthalmology Department, Dr. Rajendra Prasad Centre for Ophthalmic Sciences, All India Institute of Medical Sciences, Delhi, India; 5 Dipartimento di Scienze Otorino-Odonto-Oftalmologiche e Cervico Facciali, Universitá degli Studi di Parma, Parma, Italy; 6 Centre for Public Health, Queen's University Belfast, Belfast, UK; 7 Faculty of Epidemiology & Population Health, London School of Hygiene & Tropical Medicine, London, UK

**Keywords:** epidemiology, genetics, lens and zonules, ascorbate

## Abstract

**Background:**

Cataract is a major health burden in many countries and a significant problem in India. While observational studies show lower cataract risk with increasing dietary or plasma vitamin C, randomised controlled trials of supplements have been negative. Genetic variants in vitamin C transporter proteins (*SLC23A1*), especially rs33972313, may provide evidence on a causal association of vitamin C with cataract.

**Methods:**

We used data from a randomly selected population-based study in people aged 60 years and above in north and south India. Of 7518 sampled, 5428 (72%) were interviewed for socioeconomic and lifestyle factors, attended hospital for lens imaging and blood collection and were subsequently genotyped for rs33972313 and rs6596473. Mixed or pure types of cataract were graded by the Lens Opacity Classification System III as nuclear (2404), cortical (494) or posterior subcapsular cataract (PSC) (1026); 1462 had no significant cataract and no history of cataract surgery and 775 had bilateral aphakia/pseudophakia.

**Results:**

rs33972313 was associated with cortical (OR 2.16; 95% CI 1.34 to 3.49, p=0.002) and PSC (OR 1.68; 95% CI 1.06 to 2.65, p=0.03) but not with nuclear cataract. In analyses of pure cataracts, associations were found only between rs33972313 and pure cortical cataracts (OR 2.29; 95% CI 1.12 to 4.65, p=0.03) and with a standardised cortical opacity score. There was no association with rs6596473 and any cataract outcomes.

**Conclusions:**

Using an established genetic variant as a proxy for lifetime ascorbate concentrations, our results support a causal association of vitamin C with cataract.

## Introduction

Evidence from in vitro and animal studies strongly supports a role for vitamin C (ascorbate) in lens protection.^1^ While observational studies generally show that cataract risk is reduced with increasing dietary vitamin C intake or blood ascorbate concentration, randomised controlled trials (RCT) of supplements have not been confirmatory.^2^ These studies have been conducted mainly in high-income populations with adequate dietary intakes of vitamin C and plasma ascorbate concentrations. In two population-based studies in India with low ascorbate concentrations, we also found plasma ascorbate was inversely associated with cataract.^3 4^ These studies were cross-sectional and associations might reflect reverse causation or confounding by poverty-associated factors. A RCT in a rural population in India found no benefit in the progression of opacities over a 5-year period from supplementation with high-dose vitamins A, C and E.^5^ The lack of convincing evidence from this and other RCTs is not surprising since a priori it is unlikely that vitamin supplements given for a relatively short duration would prevent an age-related disease. Genetic variants in sodium-dependent vitamin C transporter proteins (SVCTs), *SLC23A1* and *SLC23A2* 6 are not subject to these concerns and can provide stronger evidence on the unconfounded association of ascorbate with cataract (Mendelian Randomisation (MR)). In particular, variant rs33972313 (*SLC23A1*) has demonstrated large effects on ascorbate concentrations and is considered a marker of lifelong ascorbate levels suitable for MR studies.^7 8^ In a small study of 60 patients undergoing cataract surgery in India, we reported that *SLC23A*1 (rs6596473) and *SLC23A2* (rs12479919, rs1279683) variants influenced plasma, aqueous and lens ascorbate concentrations.^9^ Since it is not possible to conduct similar investigations of ascorbate concentrations in the aqueous and lens of people without cataract, no conclusions can be drawn on whether these variants are associated with cataract risk. We report the results of an investigation using data already collected in the population-based cross-sectional study, India age-related eye disease study (INDEYE).

## Methods

### INDEYE study population

The aims of the INDEYE study were to investigate the prevalence and risk factors for age-related macular degeneration (AMD) and cataract. The study sampling has been described.^10^ In brief, people aged 60 and above were identified from household enumeration of randomly sampled villages and small towns in Haryana state (north India centre) and Tamil Nadu (south India centre), served by the participating eye hospitals (Dr Rajendra Prasad Center of Ophthalmic Sciences (RPC), Delhi and the Aravind Eye Hospital (AEH), Pondicherry). All enumerated people aged 60 and above were invited to take part.

### Ethics statement

Participants gave full informed written consent. Illiterate subjects had the information leaflet read to them and provided a thumb impression. The study complied with the Declaration of Helsinki guidelines. Ethics approval was received from the Indian Council for Medical Research, Research Ethics Committees of the All India Institute of Medical Sciences, Aravind Eye Hospital, London School of Hygiene and Tropical Medicine and Queen’s University Belfast.

### INDEYE study methods

Data collection took place between September 2004 and December 2006. Enumerators collected household and individual sociodemographic and economic data. Fieldworkers interviewed participants at home with a structured questionnaire which included tobacco and alcohol use, current and past outdoor work and duration and type of cooking fuels. Diet was assessed by 24 hours recall. Within a week of the interview, participants attended the base hospital for the clinical examination which included anthropometry (height, weight and mid-upper arm circumference (MUAC)), blood pressure, an eye examination and blood sample collection.

### Assessment of cataract

Following pupillary dilatation to at least 6 mm, digital slit beam images were taken according to a standardised protocol using the Topcon SL-D7 Digital photo slit lamp for nuclear opacities (Topcon, Tokyo, Japan). Retroillumination images of the lens (one focused on the anterior and one focused on the posterior capsule) were taken with the Neitz CT-S digital camera (Kowa Optimal, Torrance, California, USA) to capture cortical and posterior subcapsular cataract (PSC). Lens opacities were graded according to the Lens Opacities Classification System III (LOCS III)^11^ in 0.1 unit steps for each opacity up to a maximum of 6.9 for nuclear opacities and 5.9 for cortical and PSC. The training and quality assurance of the photographers and graders have been described.^10^ We categorised the type of unoperated cataract based on the LOCS III grade in the worse eye of: ≥4 for nuclear cataract, ≥3 for cortical cataract and ≥2 for PSC. The comparator group were those with no cataract (ie, opacity score of <4 nuclear and <3 cortical and <2 PSC, no dense opacities and no aphakia/pseudophakia). We chose these cut points to have high sensitivity for visually significant cataract and a marker of the severity of lens opalescence. The prevalence of cataract and cataract types has been published previously.^10^

### Blood sample

Full details have been provided.^4^ For plasma ascorbate, we collected an EDTA blood sample which was centrifuged at 4°C, stabilised with metaphosphoric acid, aliquoted and transferred to a −70°C freezer. Samples were shipped in dry ice to Queen’s University Belfast for analysis and stored at −80°C. Ascorbate was measured by automated fluorometric assay on a Cobas FARA centrifugal analyzer (Roche Diagnostics, Switzerland). The limit of detection of the assay was 2 µmol/L. Assays were standardised against the US National Institute of Standards and Technology standard reference materials. We collected a non-fasting sample of capillary blood which was assessed for glucose using a reagent strip test and reflectance metre. Buffy coats were stored at −80°C at Aravind Medical Research Foundation, Madurai, until DNA extraction between 2008 and 2009. Genomic DNA was extracted from peripheral blood leucocytes using Quiagen kits.

### Methods for present study

#### Genotyping

Genotyping was undertaken at Aravind Medical Research Foundation using a TaqMan assay in an ABI (Applied Biosystems) 7900 HT Fast Real-Time PCR system. We genotyped rs33972313 in *SLC23A1* as the primary single nucleotide polymorphism (SNP) of interest. We also genotyped *SLC23A1* SNPs rs42577623, rs6596473, rs10063949 and one *SLC23A2* SNP (rs12479919) which we previously showed influenced lens nucleus ascorbate.^9^ Details of the SNPs are provided in the online supplementary table 1.

10.1136/bjophthalmol-2018-312257.supp3 Supplementary data

We repeated genotyping for *SLC23A2* rs12479919, since 6% of calls were undetermined but despite a high call rate, the SNP failed Hardy Weinberg Equilibrium (HWE) (p=0.004) and is not discussed further. rs42577623, rs6596473 and rs10063949 were in high linkage disequilibrium (r^2^=0.89–0.93), and we show results for rs6596473 only.

### Statistical analysis

Statistical analysis was carried out using Stata V.14. We used the genhw command to calculate minor allele frequency (MAF) and HWE tests, a non-parametric test for trend for plasma ascorbate by genotype or a Kruskal-Wallis test for a two sample non-parametric comparison. We checked that rs33972313 was not confounded by covariates shown in our study to be associated with plasma ascorbate.^4^ We used logistic regression to examine the associations with type of cataract and report adjusted (age, sex, centre) ORs and 95% CIs. In sensitivity analyses, we investigated (1) associations for pure cataract type (ie, excluding mixed cataracts) and (2) the lens opacity score for each type of opacity using a standardised z-score transformation in linear regression. Analyses of the lens opacity score used the score in the worse eye for participants with two eyes and scores from one eye for participants with unilateral aphakia/pseudophakia. Differences in effect by centre were tested using an interaction term in the regression models. Analyses took account of the sampling design by use of robust standard errors and design-adjusted Wald tests. We used a Bonferroni-adjusted critical value of 0.025 for rs6596473 as this was a secondary hypothesis.

## Results

Of 7518 enumerated people, 5428 (72%) were interviewed for socioeconomic and lifestyle factors, attended an eye examination and were subsequently genotyped for rs33972313. The mean (SD) age of participants was 67.6 (6) years, 52% (2843) were women, 73% (3938) lived in rural areas and 62% (3362) were illiterate. A half, 52% (2833), used tobacco; <1% of women and 40% (1029) of men drank alcohol regularly. Overall, 16% (887) were overweight or obese and 15% (806) had MUAC indicating moderate to severe malnutrition. The distribution of plasma ascorbate was right skewed; 30% of samples were classified as 2 μmol/L, the lowest detection level of the lab, and a further 28% had concentrations less than 11 μmol/L; fewer than 20% had adequate values (>28 µmol/L) (online supplementary figure 1). Mixed or pure types of cataract were graded as nuclear (2404), cortical (494) or PSC (1026); 1462 had no significant cataract and no history of cataract surgery and 775 had bilateral aphakia/pseudophakia.

10.1136/bjophthalmol-2018-312257.supp1 Supplementary data

HWE p values and MAFs for rs33972313 were 0.12 and 0.015 and for rs6596473, 0.36 and 0.449, respectively. In analyses with rs33972313, we combined AA (n=3) with GA (n=159) due to the small numbers with the AA genotype. There was no difference in median (IQR) ascorbate concentrations by rs33972313 genotype: GG 7.3 (2–21.4) compared with GA plus AA, 6.3 (2–19.7), p=0.4. For rs6596473, the median per-genotype difference in ascorbate concentration was around 1 μmol/L, p<0.01. There was no association of rs33972313 with ascorbate-related covariates and potential confounders of the association with cataract including sociodemographic and economic characteristics, clinical and environmental factors (online supplementary table 2). In analyses of mixed cataracts, we found no association of rs33972313 with nuclear cataract and an association with cortical and PSC (table 1). In sensitivity analyses, associations were found between rs33972313 and pure cortical cataracts (OR 2.29; 95% CI 1.12 to 4.65, p=0.023) and with the standardised cortical opacity score (table 1). Addition of plasma ascorbate or other covariates to the analyses did not change the results. No associations by genotype or additive associations were found between rs6596473 and any cataract outcomes or sensitivity analyses (table 2). We checked whether our results were confounded by AMD.^12^ Results excluding 53 late AMD cases and 1240 early AMD cases were almost identical to results in table 1; p values were slightly reduced due to the smaller numbers. The ORs (95% CIs) for the association of rs33972313 were nuclear cataract: 1.38; 95% CI 0.77 to 2.47, p=0.28; cortical cataract: 2.84; 95% CI 1.45 to 5.63, p=0.003; PSC: 1.79; 95% CI 0.95 to 3.35, p=0.07.

10.1136/bjophthalmol-2018-312257.supp2 Supplementary data

**Table 1:** Association of rs33972313 with types of cataract (1) mixed cataracts, (2) pure cataracts and (3) lens opacity scores in the two centre India age-related Eye Disease population study (INDEYE)

Mixed cataracts*†	Any nuclear	Any cortical	Any posterior subcapsular
	OR (95% CI)‡	OR (95% CI)‡	OR (95% CI)‡
Cases (n)	2295	494	1026
Case genotype (n)	73 GA/AA, 2222 GG	20 GA/AA, 474 GG	37 GA/AA, 989 GG
AA and GA versus GG	1.27 (0.83 to 1.95)	2.16 (1.34 to 3.49)	1.68 (1.06 to 2.65)
P effect	0.279	0.002	0.03
P difference by centre	0.789	0.569	0.676

Pure cataracts§†	Pure nuclear	Pure cortical	Pure posterior subcapsular
	OR (95% CI)‡	OR (95% CI)‡	OR (95% CI)‡
Cases (n)	1429	192	222
Case genotype (n)	41 GA/AA, 1388 GG	9 GA/AA, 183 GG	6 GA/AA, 216 GG
AA and GA versus GG	1.11 (0.69 to 1.80)	2.29 (1.12 to 4.65)	1.19, (0.56 to 2.52)
P effect	0.671	0.023	0.653
P difference by centre	0.672	0.995	0.712

Opacity score	Nuclear score	Cortical score	Posterior subcapsular score
	Beta (95% CI)¶	Beta (95% CI)¶	Beta (95% CI)¶
Participants (n)	4318	4336	4334
Participants genotype	134 GA/AA, 4184 GG	134 GA/AA, 4202 GG	134 GA/AA, 4200 GG
AA and GA versus GG	0.007 (−0.159 to 0.173)	0.187 (0.005 to 0.369)	0.103 (−0.080 to 0.286)
P effect	0.932	0.045	0.264
P difference by centre	0.455	0.798	0.220

Any cataract type (pure or mixed) defined as Lens Opacity Classification System III grade: nuclear opacities ≥4, cortical opacities ≥3, posterior subcapsular opacities ≥2.

Comparator group (controls) without cataract or operated cataract (ie <4 for nuclear opacities and <3 for cortical opacities and <2 for posterior subcapsular cataract, no dense opacities and no aphakia/pseudophakia); n=1462, 40 GA/AA and 1422 GG.

OR and 95% CI calculated from logistic regression adjusted for age, sex, study centre.

Pure cataract type defined as Lens Opacity Classification System III grade: nuclear opacities ≥4, cortical opacities ≥3, posterior subcapsular opacities ≥2, and no other type of opacity.

Beta coefficient (Beta) and 95% CI from regression analysis adjusted for age, sex, study center.

Standardised scores of Lens Opacity Classification System III grade: nuclear opacity score in the worse eye graded in 0.1 units from 0.5 to 6.9; cortical opacity score in the worse eye graded in 0.1 units from 0 to 5.9; posterior capsular opacity score in the worse eye graded in 0.1 units from 0 to 5.9.

**Table 2:** Association of rs6596473 with types of cataract (1) mixed cataracts, (2) pure cataracts and (3) lens opacity scores in the two centre India age-related Eye Disease population study (INDEYE)

Mixed cataracts*†	Any nuclear	Any cortical	Any posterior subcapsular
	OR (95% CI)‡	OR (95% CI)‡	OR (95% CI)‡
Cases (n)	2288	491	1026
Additive association	1.05 (0.97 to 1.15)	1.02 (0.86 to 1.22)	1.05 (0.93 to 1.19)
P effect	0.220	0.789	0.406
P difference by centre	0.826	0.782	0.918

Pure cataracts§†	Pure nuclear	Pure cortical	Pure posterior subcapsular
	OR (95% CI)‡	OR (95% CI)‡	OR (95% CI)‡
Cases (n)	1421	187	218
Additive association	1.05 (0.95 to 1.17)	0.97 (0.76 to 1.24)	0.99 (0.78 to 1.27)
P effect	0.304	0.825	0.966
P difference by centre	0.904	0.564	0.821

Opacity scores¶	Nuclear score	Cortical score	Posterior subcapsular score
	Beta (95% CI)**	Beta (95% CI)**	Beta (95% CI)**
Participants (n)	4292	4310	4308
Per allele association	0.030 (−0.006 to 0.065)	−0.018 (−0.054 to 0.019)	0.021 (−0.022 to 0.063)
P effect	0.103	0.339	0.338
P difference by centre	0.683	0.181	0.761

Any cataract type (pure or mixed) defined as Lens Opacity Classification System III grade: nuclear opacities ≥4, cortical opacities ≥3, posterior subcapsular opacities ≥2.

Comparator group (controls) without cataract or operated cataract (ie <4 for nuclear opacities and <3 for cortical opacities and <2 for posterior subcapsular cataract, no dense opacities and no aphakia/pseudophakia); n=1462.

OR and 95% CI calculated from logistic regression adjusted for age, sex, study centre.

Pure cataract type defined as Lens Opacity Classification System III grade: nuclear opacities ≥4 cortical opacities ≥3, posterior subcapsular opacities ≥2 and no other type of opacity.

Standardised scores of Lens Opacity Classification System III grade: nuclear opacity score in the worse eye graded in 0.1 units from 0.5 to 6.9; cortical opacity score in the worse eye graded in 0.1 units from 0 to 5.9; posterior capsular opacity score in the worse eye graded in 0.1 units from 0 to 5.9.

Beta coefficient (Beta) and 95% CI from regression analysis adjusted for age, sex, study centre.

## Discussion

We found an approximately twofold association of rs33972313 with both mixed and pure cortical cataracts and with mixed PSC cataracts. The association with cortical cataract was also found using the continuous cortical opacity score. rs33972313 in exon 8 is a missense *SLC23A1* variant causing a valine-to-methionine substitution in SVCT1 and has been shown experimentally to reduce ascorbate transport by 40%–50%.^13^ The main function of SVCT1, expressed primarily in the small intestine, liver and kidney, is absorption of dietary vitamin C and renal reabsorption of ascorbate in the circulating blood.^14^ SVCT1 therefore plays a key role in the regulation of whole body ascorbate homeostasis. Of *SLC23A1* polymorphisms, rs33972313 has been consistently shown to influence plasma ascorbate concentrations.^7 8^ A meta-analysis of five studies, over 15 000 participants, in the UK reported lower ascorbate concentration (–5.98, 95% CI −8.23 to −3.73) per A allele; ascorbate levels were generally high, around 45–55 µmol/L in four studies and lower (30 µmol/L) in one study with a smaller allele difference in ascorbate of 2.87 lower.^7^ In the Copenhagen General Population Study, the mean ascorbate levels in the GG and GA genotypes were 29 and 26 µmol/L and 22 µmol/L in the four AA carriers.^8^ It is noticeable that in these European studies, the per-allele differences were smaller (around 3 µmol/L) in the two studies with mean ascorbate concentrations of 30 µmol/L compared with per-allele differences of 6 µmol/L in the four studies with higher mean ascorbate levels. In our study, we found smaller differences in ascorbate concentrations by genotype reflecting lower ascorbate concentrations. Our results were limited by the high proportion (30%) with ascorbate concentrations at the limit of detection and our relatively small sample given the low MAF of rs33972313. For *SLC23A1* variant rs6596473, the UK meta-analysis reported per-allele mean differences of 3 µmol/L in the discovery study replicated with smaller differences of 1 µmol/L.^7^ We found a similar per-allele difference in ascorbate concentrations but no associations between rs6596473 and any cataract outcomes.

In our study, rs33972313 was not associated with other determinants of ascorbate, consistent with previous studies.^7 8^ These properties of rs33972313—large per-allele effects on ascorbate, lack of confounding and pleiotropy—account for its choice as a key SNP for MR analyses of ascorbate-related outcomes.^8 15 16^

### Study strengths and limitations

We used an established cataract grading scale, LOCS III^11^ and chose cut points to categorise type of cataract similar to other studies (reviewed in our cataract prevalence paper).^10^ The validity of our cataract definition for genetic studies is supported by other results from the INDEYE study, for example, an association of *EPHA2* variants with cortical but not nuclear cataract^17^ in agreement with results from other studies.^18^ Our sensitivity analysis showed that the findings for rs33972313 and cortical cataract were robust to different cataract measures. Our study was based on a random population sample with an over 70% response. However, our study was relatively small given the low MAF of rs33972313 (0.015). While the rs6596473 MAF was larger (0.45), the small per-allele influence of this SNP on ascorbate suggests larger studies than ours would be required.

We had only a single measurement of plasma ascorbate taken in later life. We found that adjustment for plasma ascorbate did not attenuate the association with rs33972313 possibly reflecting measurement error in ascorbate and that current concentrations may not reflect ascorbate concentrations at earlier life stages. Plasma vitamin C levels in our study were low. The few studies that have measured plasma vitamin C levels in India, including our previous studies,^3 9^ also reported low levels.^19 20^

SVCT1 has not been found to be expressed in ocular tissues^21^ or expressed only weakly in human lens epithelial tissues.^22^ SVCT2 is expressed in nearly all cell types including the ciliary,^22^ corneal^21 23^ and lens epithelium.^22 24^ We originally aimed to investigate the SVCT2 variant (*SLC23A2*, rs12479919) but this was not possible because of genotyping errors. While findings for rs12479919 and cataract risk would undoubtably provide additional information on the role of genetic variation in SVCT transporters, we do not consider that the lack of this information limits the results we report for *SLC23A1* variant rs33972313. Unlike most variants in *SLC23A1* or *SLC23A2,* the molecular consequences of rs33972313, encoding an amino acid change in the SVCT1 protein, are known. We hypothesised that individuals carrying the risk allele of this variant would have a higher risk of cataract due to genetically lower plasma ascorbate. Full understanding of the influences and mechanisms for upregulation and transport of plasma ascorbate in human eyes is lacking.^22^ Many studies have used rodent or rabbit tissues and findings may not apply to humans. Ma *et al* recently showed expression of SVCT2 varied between mouse and human ocular tissues.^22^ The authors found SVCT2 to be highly expressed in human ciliary pigmented epithelium located near the ciliary stromal microvasculature,^22^ a finding consistent with upregulation of ascorbate achieving molar concentrations in the aqueous. The concentration of ascorbate in the blood is a very strong predictor of the level in the aqueous, as demonstrated by two studies, one in the USA^25^ and our study in India.^9^ Both studies showed log-linear associations of plasma ascorbate with aqueous ascorbate. We also found genotype differences in aqueous ascorbate for *SLC23A1* rs6596473 and in lens nucleus ascorbate with *SLC23A2* rs12479919. In these analyses, plasma ascorbate was the strongest influence on concentrations of aqueous and lens ascorbate. Genotype differences in plasma ascorbate were also found for both *SLC23A1* and *SLC23A2* variants suggesting that *SLC23A2* may influence plasma ascorbate in tissues outside the eye. The only other published results on a *SLC23A2* variant (rs1279683) in an eye condition also found a genotype difference in plasma ascorbate and that rs1279683 and plasma ascorbate were associated with an increased risk of primary open glaucoma.^26 27^ The authors concluded that the association of rs1279683 with glaucoma was directly mediated through plasma ascorbate.

The plausibility of our findings relates to the importance of ascorbate in lens protection, especially against the damaging effects of ultraviolet radiation B (UVB) and oxygen.^1 28^ Ascorbate plays a complex role including absorption of UVB, maintenance of the powerful antioxidant glutathione, oxygen reduction, scavenging and quenching of free radicals. The outer epithelial and cortical cells are most exposed to UVB radiation and evidence from epidemiological studies that UVB is a risk factor for cataract is strongest for cortical cataracts.^29^

In conclusion, using a robust genetic variant as a proxy for lifetime ascorbate concentrations, our results support a causal association between ascorbate and cortical cataract. Our study took place in the Indian setting with low concentrations of plasma ascorbate and it is uncertain whether our results would apply to other populations with adequate dietary vitamin C intakes and plasma ascorbate concentrations.
