# Supplementary material for: Genetic variants in a sodium-dependent vitamin C transporter gene and age-related cataract
Source: Br J Ophthalmol. 2018 Nov 15;103(9):1223–7. doi: 10.1136/bjophthalmol-2018-312257 (PMC6709767; doi:10.1136/bjophthalmol-2018-312257)
Supplement: Supplementary data [file bjophthalmol-2018-312257supp003.pdf]

Supplementary Table 1. SLC23A1/2 Single Nucleotide Polymorphisms, Location, Alleles, Molecular consequences, Minor Allele Frequencies (MAFs) and Hardy Weinberg Equilibrium tests for participants in the India age-related Eye Disease population study (INDEYE) with corresponding reported MAFs in the 1000 genomes project in South Asian and European populations

SNP[1]	Location	Major/Minor Allele	Molecular consequences	HWE[2]	MAF[3]	MAF SAS[4]	MAF EUR[5]
SLC23A1 Chromosome 5							
rs33972313	139.379.813	G/A	Exon 8 Missense variant.	0.1214	0.0150	0.0143	0.0348
rs4257763	139.378.470	G/A	Intron	0.3296	0.4754	0.4990	0.6262
rs6596473	139.374.887	G/C	Intron	0.3630	0.4488	0.4775	0.3410
rs10063949	139.383.837	T/C	Intron 2KB upstream variant	0.3994	0.4611	0.4826	0.3817
SLC23A2 Chromosome 20							
rs12479919	5.000.094	C/T	Intron	0.004	0.2826	0.2894	0.3658

[1] Single Nucleotide Polymorphism

[2] p value for departure from Hardy Weinberg Equilibrium (HWE)

[3] Minor Allele Frequency (MAF) INDEYE Study

[4] Minor Allele Frequency (MAF from 1000 genome study for South Asian ancestry available in https://www.ncbi.nlm.nih.gov/snp accessed 14 June 2018

[5] Minor Allele Frequency (MAF) from 1000 genome study for European ancestry available in https://www.ncbi.nlm.nih.gov/snp accessed 14 June 2018
