# Supplementary figures and images for: Genetic variants in a sodium-dependent vitamin C transporter gene and age-related cataract
Source: Br J Ophthalmol. 2018 Nov 15;103(9):1223–7. doi: 10.1136/bjophthalmol-2018-312257 (PMC6709767; doi:10.1136/bjophthalmol-2018-312257)

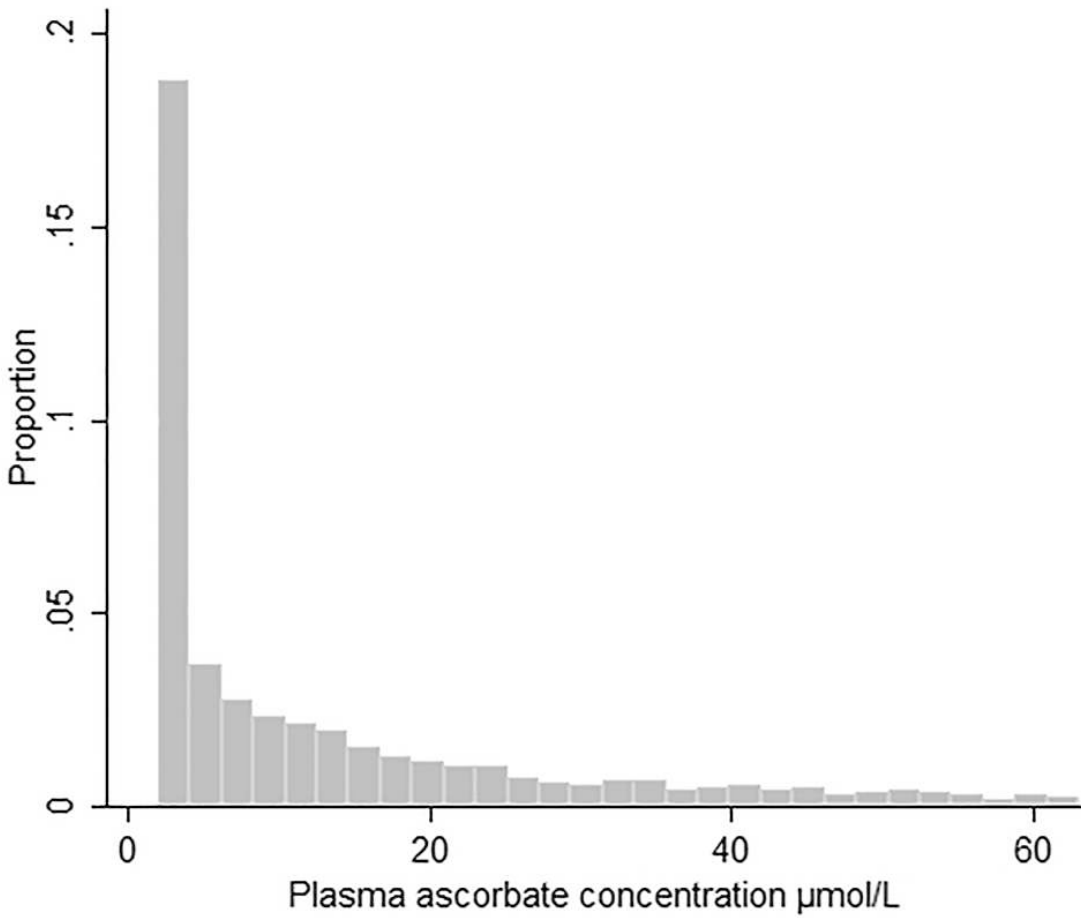

Supplement: Supplementary data [file bjophthalmol-2018-312257supp001.pdf]
