# Supplementary material for: Genetic variants in a sodium-dependent vitamin C transporter gene and age-related cataract
Source: Br J Ophthalmol. 2018 Nov 15;103(9):1223–7. doi: 10.1136/bjophthalmol-2018-312257 (PMC6709767; doi:10.1136/bjophthalmol-2018-312257)
Supplement: Supplementary data [file bjophthalmol-2018-312257supp002.pdf]

Supplementary Table 2. Characteristics of participants in the India age-related Eye Disease population study (INDEYE) by *SLC23A1* SNP rs33972313

Characteristics	***SLC23A1* SNP rs33972313**		
	**GG**<br>**n=5268**	**GA and AA[1]**<br>**n=160**	**p-value[2]**
Age, mean years (SD)	67.6 (6.5)	66.9 (5.8)	0.07
Women, n (%)	2763 (52.5)	80 (50.0)	0.61
Low Socioeconomic status[3], n (%)	1184 (22.5)	31 (19.4)	0.36
Ever use of tobacco, n (%)	3250 (61.7)	99 (61.9)	0.97
Biomass fuel use, mean years (SD)	47.5 (13.3)	46.7 (14.3)	0.64
High Sun exposure[4] n (%)	1286 (24.4)	38 (23.8)	0.86
Body Mass Index ≥25 kg/square metre, n (%)	856 (16.3)	31 (19.5)	0.30
Moderate and severe malnutrition[5], n (%)	790 (15.0)	16 (10.0)	0.07
Diabetes[6], n (%)	268 (5.1)	8 (5.0)	0.96
Cholesterol (µmol/L) mean (SD)	4.52 (1.06)	4.66 (0.96)	0.15
Diastolic Blood pressure (mm/Hg), mean (SD)	74.1 (13.3)	73.5 (14.7)	0.69

[1] 157 participants with GA genotype and 3 participants with AA genotype.

[2] p-value for difference between genotype (GG versus GA plus AA) calculated from Design-based Pearson Chi- squared test for categorical variables and Design adjusted Wald test for continuous variables.

[3] lowest quintile of the socio-economic score derived using principal component analysis (based on caste, landholding, roof type, number of rooms) and categorized by quintiles.

[4] highest quartile of midday sun exposure calculated from questionnaire responses on time spent outdoors at different over job and life periods and categorized by quartiles.

[5] Moderate and severe malnutrition defined as mid-upper arm circumference (<22 in men and <20 in women).

[6] Diabetes defined as blood glucose ≥ 200 mg/dl
